# Investigating the Antibacterial Activity of Polymeric Membranes Fabricated with Aminated Graphene Oxide

**DOI:** 10.3390/membranes11070510

**Published:** 2021-07-07

**Authors:** Muhammad Zahid, Saba Akram, Anum Rashid, Zulfiqar Ahmad Rehan, Talha Javed, Rubab Shabbir, Mahmoud M. Hessien, Mahmoud E. El-Sayed

**Affiliations:** 1 Department of Chemistry, University of Agriculture, Faisalabad 38000, Pakistan; rmzahid@uaf.edu.pk; 2 Department of Materials, National Textile University, Faisalabad 37610, Pakistan; saba.akram1980@gmail.com (S.A.); anumrashid800@yahoo.com (A.R.); 3 Department of Agronomy, University of Agriculture, Faisalabad 38000, Pakistan; mtahaj@fafu.edu.cn; 4 College of Agriculture, Fujian Agriculture and Forestry University, Fuzhou 350002, China; rubabshabbir28@gmail.com; 5 Department of Chemistry, College of Science, Taif University, P.O. Box 11099, Taif 21974, Saudi Arabia; m.hessien@tu.edu.sa; 6 Department of Food Science and Technology, Faculty of Agriculture, Tanta University, Tanta 21527, Egypt; mahmoud.elsayed@agr.tanta.edu.eg

**Keywords:** aminated graphene oxide, membrane technology, phase inversion, antibacterial

## Abstract

A novel, functionalized graphene oxide–based cellulose acetate membrane was fabricated using the phase inversion method to improve the membrane characteristics and performance. We studied the effect of aminated graphene oxide (NH_2_–GO) composite on the CA membrane characteristics and performance in terms of membrane chemistry, hydrophilicity, thermal and mechanical stability, permeation flux, and antibacterial activity. The results of contact angle and water flux indicate the improved hydrophilic behavior of composite membranes in comparison to that of the pure CA membrane. The AGO-3 membrane showed the highest water flux of about 153 Lm^−2^h^−1^. The addition of hydrophilic AGO additive in CA membranes enhanced the antibacterial activity of AGO–CA membranes, and the thermal stability of the resulting membrane also improved since it increases the Tg value in comparison to that of a pristine CA membrane. The aminated graphene oxide (NH_2_–GO) was, therefore, found to be a promising additive for the fabrication of composite membranes with potent applications in wastewater treatment.

## 1. Introduction

One of the prime challenges in the sustainment of modern society is securing adequate resources of water with desired properties for different designated uses. Therefore, an important role is expected to be played by membrane technology in crucial areas, i.e., seawater and brackish water desalination, drinking water, and wastewater and its reuse, owing to its advantageous features such as efficient and selective separation, handling ease, eco-friendly nature, and stability in water treatment [1,2].

Membrane-based separation processes mostly rely on polymeric materials due to their high perm-selectivity, greater flexibility, chemical stability, mechanical strength, and easy film forming property [3]. Cellulose acetate (CA), one of major carbohydrate polymers, is widely utilized as a membrane material and is well suited for broad-spectrum filtration. It possesses several properties such as potential flux, increased biocompatibility, and moderate hydrophilicity. Despite these advantages, cellulose acetate is poorly resistant toward fouling [4,5]. Since most of polymeric membranes are either hydrophobic in nature or they possess less hydrophilicity, they face the main issue of biofouling on their surface, which limits their working life [6].

Membrane biofouling is the most ubiquitous among fouling types and is recognized as the deposition, attachment, and proliferation of microbial communities through the release of EPS (extracellular polymeric substances), which results in the formation of a biofilm on the membrane surface. Biofilm aids in concentration polarization on the surface of a membrane, the consequences of which are blockage of pores, reduction in permeates flux, salt rejection, and rise in transmembrane pressure, which then requires more energy expenditure and time to overcome it [7,8,9]. Most of the organic pollutants present within feed solutions lower the ability of membrane separation via agglomeration upon the surface or inside the pore structure of the membrane [10]. Hence, the fabrication of nanocomposite membranes with enhanced hydrophilicity, permeate flux, and anti-biofouling performance is one of the common strategies in the mitigation of biofouling issues [11]. 

Nanocomposite membranes, therefore, emerged as a new class in providing a promising solution against these limitations of polymeric membranes and to meet specific applications of water treatment. Nanocomposite membranes are generated through the blending of nanomaterials within a polymeric matrix. Nanomaterials within hybrid membranes help out in the tuning of the polymer structure and enhancing physiochemical properties such as hydrophilicity, porosity, charge density, and thermal and mechanical stability along with the introduction of unique functionalities, i.e., antibacterial, adsorptive, or photocatalytic activities [12,13,14]. The nanomaterials employed commonly in polymer membranes are alumina (Al_2_O_3_), titanium dioxide (TiO_2_), zinc oxide (ZnO), silica (SiO_2_), CNT, Fe_3_O_4_, zirconia (ZrO_2_), and graphene oxide (GO) [15,16,17].

Graphene oxide (GO) is an outstanding nanomaterial with oxygen-containing functional groups, such as hydroxyl, carboxyl, carbonyl, and epoxy groups, which provide hydrophilicity and antibacterial properties to GO. It exhibits high strength, high aspect ratio, planar structure, and easy capability of surface functionalization. However, since GO has an amphiphilic nature, it restricts the hydrophilic nature of it within composite membranes [18,19]. GO has been widely used as membrane material because it improves the membrane characteristics such as hydrophilicity, adsorption, and water flux as reported in literature. Chemical functionalization of GO can be easily done, and different graphene derivatives such as sulfonated graphene, fluorinated graphene, hydrogenated graphene, and aminated graphene are emerging as a new generation. Incorporation of these above-mentioned functional groups to the graphene oxide structure has the ability to improve the solubility or dispersibility as well as the reaction with polymers and biological and organic compounds. The introduction of an amino group into the carbon network, such as graphene oxide, graphene, carbon nanotubes, etc., has enabled applications as nanofillers in photoluminescent materials, metal-free catalyst, polymer coatings, and composites. Amino group introduction also enhanced the antimicrobial characteristics of that material [20]. Li et al. fabricated a polyamide membrane embedded with aminated reduced graphene oxide, and they found enhanced water flux, high antibacterial activity, and a high rejection rate of different salts [21]. In another study, researchers prepared aminated graphene oxide nanohybrid UF membranes, and their results revealed that the water flux of nanohybrid membranes was three folds higher than that of a pristine membrane. Additionally, these membranes have higher fouling resistance [22]. 

In this present research work, we fabricated an aminated graphene oxide–based cellulose acetate membrane using the conventional NIPS method. The separation properties of fabricated membranes were systematically analyzed in terms of antibacterial activity, mechanical strength, and water flux. Other properties such as hydrophilicity and thermal stability were also determined and discussed.

## 2. Materials and Methods

### 2.1. Materials and Synthesis Protocol

Cellulose acetate (CA) of Mw = 50 kDa in powder form was purchased from Sigma Aldrich Germany. Before use, CA powder was dried in a vacuum oven at 105 °C. Tetrahydrofuran (THF, 99.5%), sodium nitrate (NaNO_3_), ethylene glycol (99.5%), sulfuric acid (98.5%), KMNO_4_ (99.7%), graphite powder (99.99%), and NH_4_OH were purchased from Sigma-Aldrich. Ultrapure water (Purelab, Elga, UK) was used for the preparation of the solutions. Unless specified, all of the reagents were used as received without further purification.

Graphene oxide was synthesized by following the modified Hummers method. Graphite powder (5 g) was taken in a 500 mL flask together with 2.5 g of sodium nitrate. The flask was put in an ice bath, and 250 mL of sulfuric acid was added into the flask followed by vigorous stirring. Next, 30 g potassium permanganate (KMnO_4_) was added gradually into the solution over 2 h with continuous stirring in an ice bath. Then the ice bath was removed and, for the completion of oxidation process, the suspension was stirred continuously for 4 days at 30–35 °C. About 200 mL of distilled water was added and the solution was heated at 98 °C temperature with continuous stirring. Finally, the solution was cooled down, and 30 mL of hydrogen peroxide was added, which reacted with the excess of potassium permanganate. Centrifugation was done to separate the graphene oxide. Then, GO was washed several times with distilled water for the removal of impurities. After washing, GO was dried in oven at 60 °C for 20 h.

**Figure 1:**
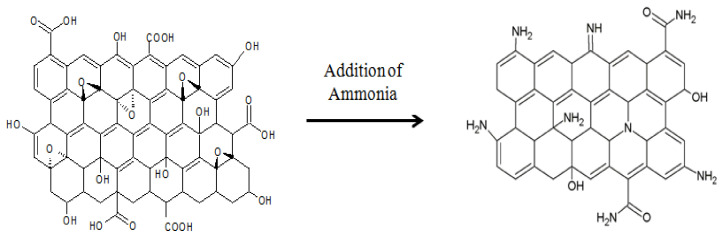
Structure of aminated graphene oxide after reaction.

**Figure 2:**
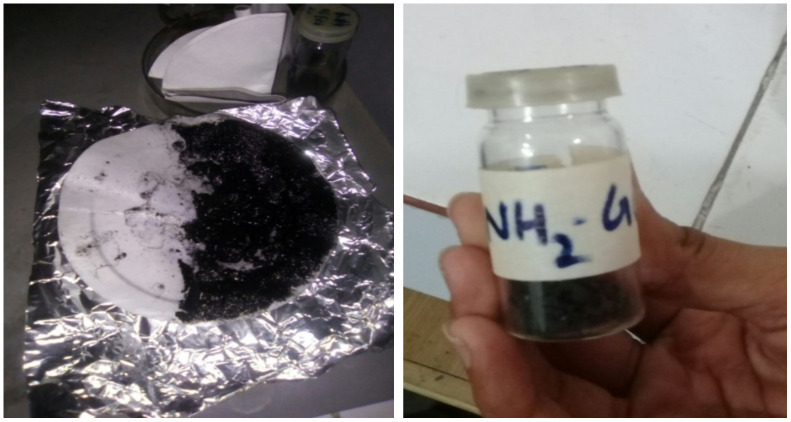
Synthesized aminated graphene oxide (NH_2_–GO).

Aminated graphene oxide composite was synthesized by solvothermal reaction of graphene oxide with ethylene glycol and ammonia in autoclave reactor (Figure 1). Then, 1 g of synthesized GO was sonicated in 20 mL distilled water followed by the addition of 220 mL ethylene glycol in the beaker. Further, 6 mL of ammonia solution was added into the solution and transferred to the autoclave for solvothermal reaction at 150 °C for 15 h. After the completion of reaction, filtration of the precipitate was done, and the precipitate was washed with distilled water several times. Finally, the precipitate was kept in an oven to dry at 60 °C for 20 h (Figure 2).

NH_2_–GO-based CA membranes were fabricated by conventional phase inversion method. Firstly, NH_2_–GO was dispersed in tetrahydrofuran under ultrasonication for 10 h. The casting solution was prepared by dissolving 15% cellulose acetate into tetrahydrofuran solvent. Precise amount of NH_2_–GO was added into the CA solution under magnetic stirring to homogenize the solution. For the removal of air bubbles, the prepared casting solution was left overnight at room temperature. The resultant solution was cast on a glass plate with the help of a self-made casting knife. Then the casting membrane was immersed in a nonsolvent coagulation bath containing distilled water for 20 h to induce phase inversion. After some minutes, the membrane was detached from the glass plate and sandwiched between sheets of filter paper to dry at room temperature. The composition of the casting solution with different concentrations of NH_2_–GO is given in Table 1.

### 2.2. Characterization

The structure of the top membrane surface was characterized by field emission scanning electron microscopy (FEI, Quanta FEG 450). Membrane samples were attached to the grid using copper tape and sputtered with gold by means of a sputter coater (Quorum Q150R ES, Quorum technologies Ltd., Ashford, Kent, England). The crystal structure of the AGO membranes was characterized by PANalytical X’pert X-ray diffraction (XRD).

Fourier-transform infrared spectroscopy (FTIR) having a wave range of 600–4000 cm^−1^ was used to analyze the chemical composition and structure of the membrane surface. Surface morphology of the fabricated membranes was determined by scanning electron microscopy. Hydrophilicity of the fabricated membrane surface was analyzed via an Attension Theta Tensiometer using a well-known method named as water contact angle measurement. To measure the contact angle, water was used as the probe liquid. Through a goniometer device, the static contact angle (formed between the water drop and the membrane surface) was measured by the sessile drop method at 25 °C. The contact angle was reported via taking the average of five different measurements taken at different locations upon the membrane surface.

An Instron tensile test machine was used to test the mechanical properties of the hybrid membranes. Before testing, membranes samples were cut according to a standard shape at 25 °C. A differential scanning calorimeter (DSC), model DSC 250 from TA instruments, USA, was used to determine the thermal properties of AGO membranes. Fabricated membranes of 10 mg were sealed in a pan made up of aluminum, and experiments were carried out under an N_2_ atmosphere at temperatures ranging from 25 to 300 °C, with at heating rate of 10 °C/min.

### 2.3. Membrane Water Permeability and Antimicrobial Activity

The membrane water permeability of composite membranes was evaluated by a cross-flow disc holder holding 50 cm^2^ of effective membrane area. Membranes were soaked for 3 h in ultrapure water prior to permeability measurements. Compaction of membranes was done initially for 1 h at a transmembrane pressure of 5 bar. Measurements of permeability were conducted at room temperature with varying pressures of 1–5 bar and a constant cross-flow of 1 L/min [23].

The water flux was defined by the following formula:

$$J_{w}=\frac{V}{A\times t}$$

where *J_w_* is the pure water flux in Lm^−2^h^−1^, *V* is known as the permeate volume in L, *A* is the membrane area in m^2^, and *t* is the filtration time in h.

The inhibition zone method was employed for evaluating the antibacterial activity of CA–AGO composite membranes using the agar well diffusion method. In this procedure, a plate containing agar medium was inoculated with a microbial strain, i.e., *E. coli* to ensure its growth. Then, a 6–8 mm diameter hole was punched aseptically at six different positions using a cork borer followed by introduction of extract (about 20 µL) into each well and a control drug (i.e., ampicillin) at the central hole with a syringe already containing the testing microbe. The agar plates were then incubated under suitable conditions depending upon the testing microbe. This resulted in diffusion within the agar medium of an antimicrobial agent that inhibits the growth of *E. coli*. The inhibition zone thus formed around each well containing the extract and test microbe was measured.

## 3. Results and Discussion

**Figure 3:**
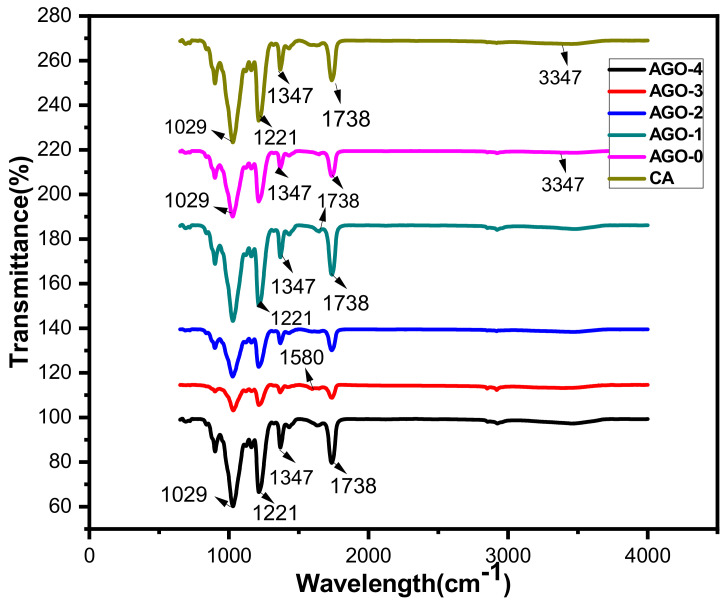
FTIR spectrum of AGO membranes.

The FTIR spectrum for GO membranes as shown in Figure 3 exhibited stretching vibrations at 1029 cm^−1^ that indicate the presence of the epoxy group C–O, while peaks at 1221 and 3347 cm^−1^ may be attributed to the stretching vibration of aromatic carbon C=C and hydroxyl groups –OH, respectively. Additionally, the carbonyl group C=O was also present in the graphene oxide, and the peak at 1738 cm^−1^ indicates the presence of that group in the membranes. Furthermore, AGO membranes have peaks at 1580, 1630, and 1738 cm^−1^ due to the presence of an amide group, while they exhibit stretching vibration at 1211 cm^−1^ due to the presence of an aromatic amine group N–H, which confirms the successful formation of aminated graphene oxide as mentioned in the literature [24].

**Figure 4:**
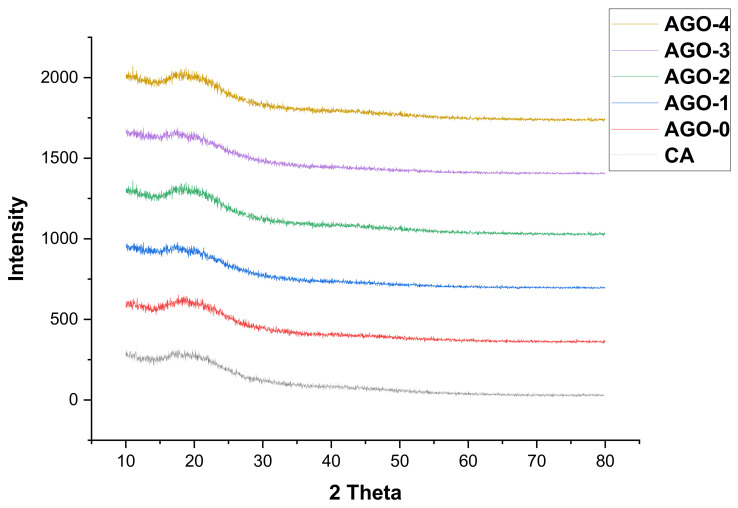
XRD of AGO membranes.

XRD analysis was performed to characterize the impact of graphene oxide loading on crystalline structure and the chain’s rearrangement of the polymer matrix. As shown in Figure 4, the semi-crystalline structure of CA was indicated by two characteristic peaks at *2θ* = 15.40° and 20.40°. It has been shown that the peak intensity increases with the addition of aminated GO (AGO). It was also observed that the XRD pattern of aminated graphene oxide synthesized membranes was very much similar to that of the pristine membrane, indicating that the AGO is well dispersed in the polymer matrix. The result can also be confirmed by SEM images [25].

**Figure 5:**
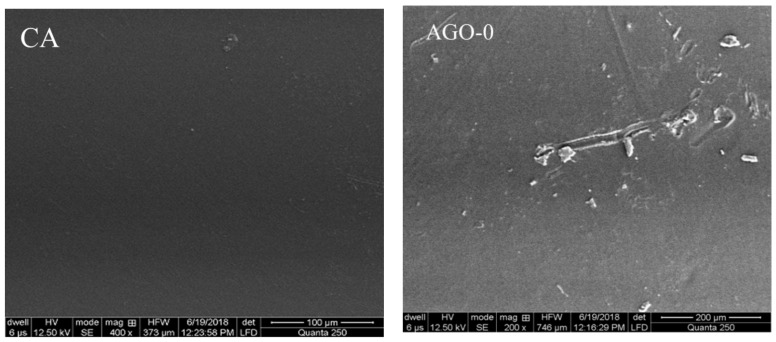

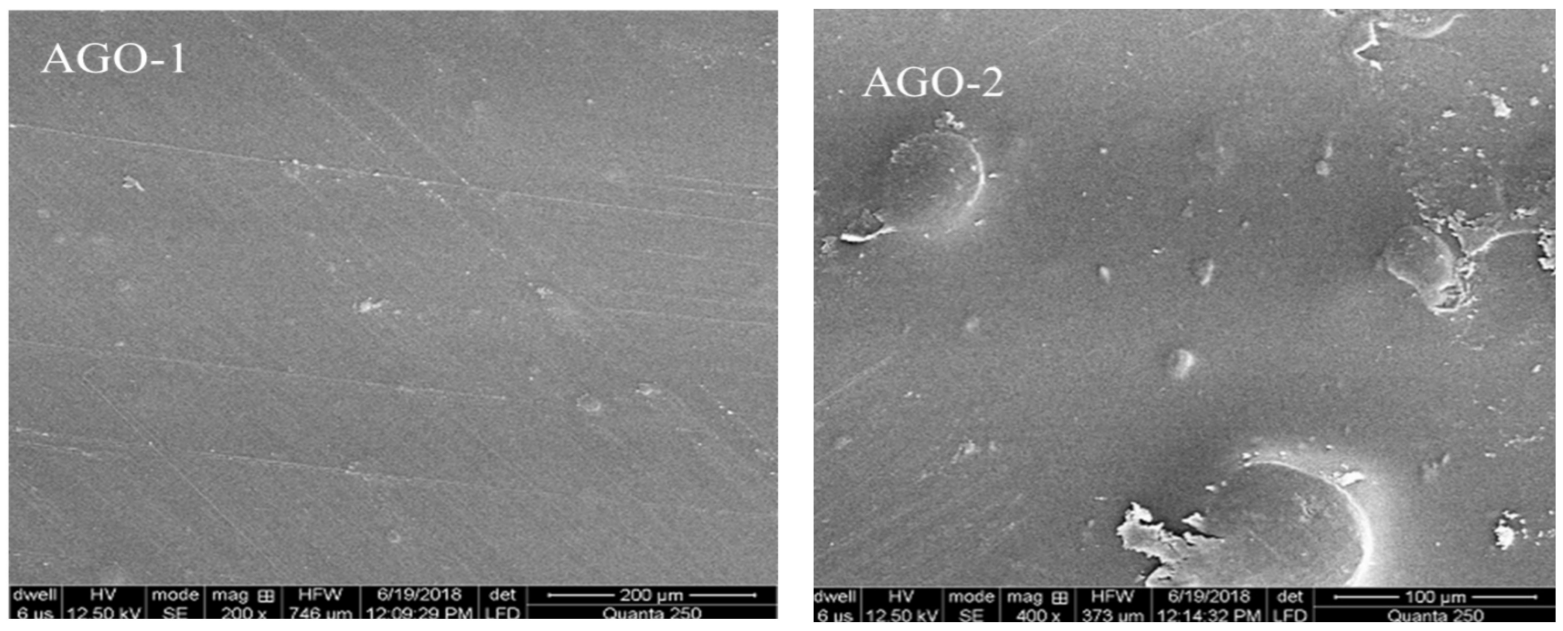
SEM images of surface morphology of AGO membranes.

Figure 5 shows the surface morphology of pristine and AGO-embedded nanocomposite membranes. The aminated GO in nanocomposite membranes is uniformly distributed as compared to that in the pristine one and is the one with only GO contents. This could be due to the excellent steric effect of GO after modification with amination [26].

**Figure 6:**
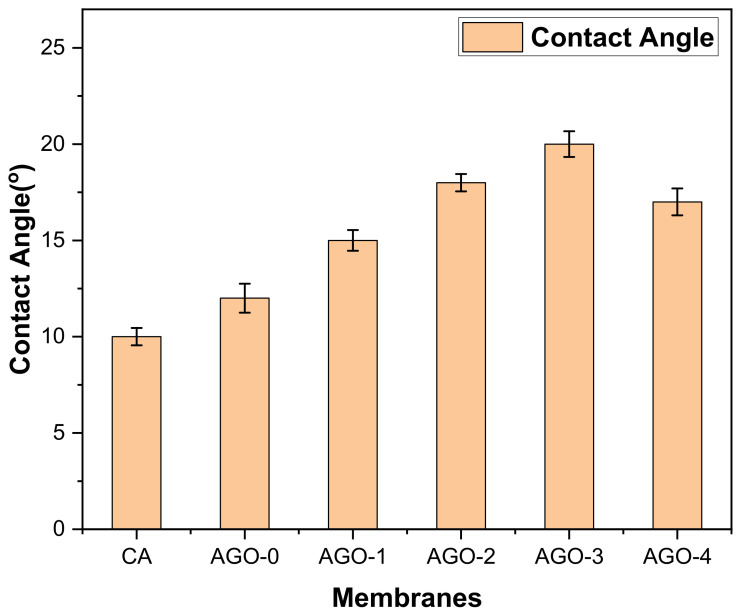
Contact angle of AGO membranes.

Hydrophilicity is another important parameter of membranes and plays an important role to improve the permeability as well as anti-biofouling properties of the membranes. During the membrane separation processes, foulants have the ability to adsorb easily on the hydrophobic membrane surface leading to fast attenuation of water permeability [27]. The surface hydrophilicity of NH_2_–GO/CA membranes was analyzed by calculating the water contact angle. The results of water contact angle measurements showed a valuable decrease with the increase of NH_2_–GO content in the hybrid membranes. This may be caused by the presence of an amine group as well as oxygen functional groups in NH_2_–GO, which are hydrophilic in nature. The loading of these fillers on the top surface of membranes during phase inversion leads to a decrease in the contact angle of membranes [28,29]. Due to the inherent hydrophilic property of CA and functionalized GO, all the modified membranes possess an extremely low contact angle of water, which confirms their excellent hydrophilicity as shown in Figure 6.

**Figure 7:**
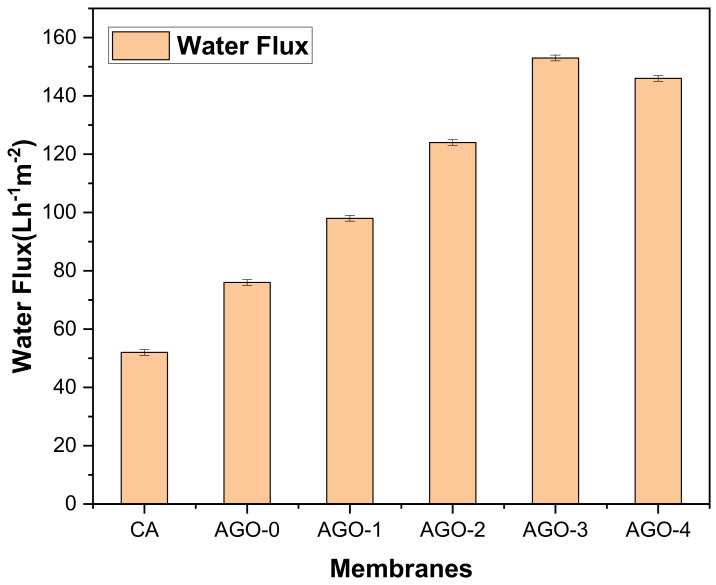
Water flux of AGO membranes.

The permeance of the fabricated membranes was evaluated by the water flux and is represented in Figure 7. The NH_2_–GO membranes showed an enhanced water permeability trend with the incorporation of NH_2_–GO composites. The AGO-3 membrane exhibited the highest water flux of about 153 Lm^−2^h^−1^. Various factors are involved in causing the enhanced water permeability of NH_2_–GO membranes. Firstly, the hydrophilicity of the fabricated membranes was improved by the incorporation of NH_2_–GO composites, which directly results in enhanced water flux. We can say that the enhancement in hydrophilicity led to an increase in water flux because of the attraction of water molecules toward the membrane matrix and helped them to pass through the membrane. Secondly, by the addition of the composite material, the packing of the polymer chain may be disrupted leading to an increase in free volume or the creation voids. Thirdly, the incorporation of composites may offer more pathways for water molecules to pass quickly through the membrane [27]. However, further addition of NH_2_–GO composites into the membrane caused a decrease in water flux. Because, during fabrication, the thickness of the casting solution increases leading to a decrease in the pores of the membranes. Li et al. fabricated reduced graphene oxide–NH_2_ polyamide membranes, and their results showed that modified GO membranes have a high water flux. This is due to the addition of R-GO–NH_2_ because the composite provides passage to the water molecules [21]. The average pore size could be assumed to be from 0.06 to 0.08 µm as a hypothetical observation by looking into the water permeability results of these nanocomposite membranes. A similar correlation of pore size distribution and water flux was observed in our previous work [30].

**Figure 8:**
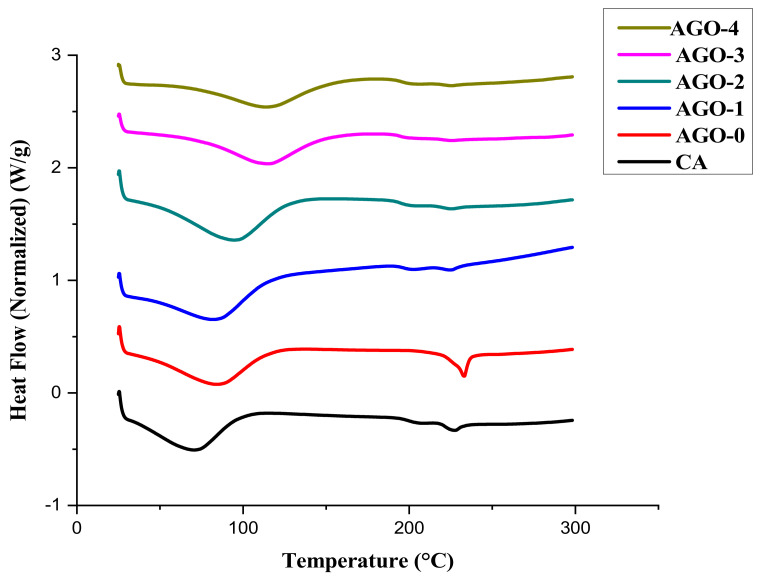
DSC of AGO membranes.

Figure 8 shows the DSC analysis of AGO membranes, giving information about the glass transition temperature. The results of DSC analysis showed different behaviors during the heating process before and after the addition of aminated graphene oxide composites in membrane. The pure cellulose acetate shows a glass transition temperature (Tg) within the 55–60 °C range, which increased with the addition of aminated GO within the CA matrix. The temperature was shifted toward the higher temperature region (70 to 150 °C) and indicated the improved thermal stability of the NH_2_–GO composite. It can be concluded that the addition of composite gave extra or more stability to the membranes, which in turn led to a shift in temperature. However, with further addition of the NH_2_–GO composite, the thermal stability of the AGO membrane decreased. It might be possible that the bonding between the polymer chain and composite is not strong and more addition of composite weakens the interactions.

**Figure 9:**
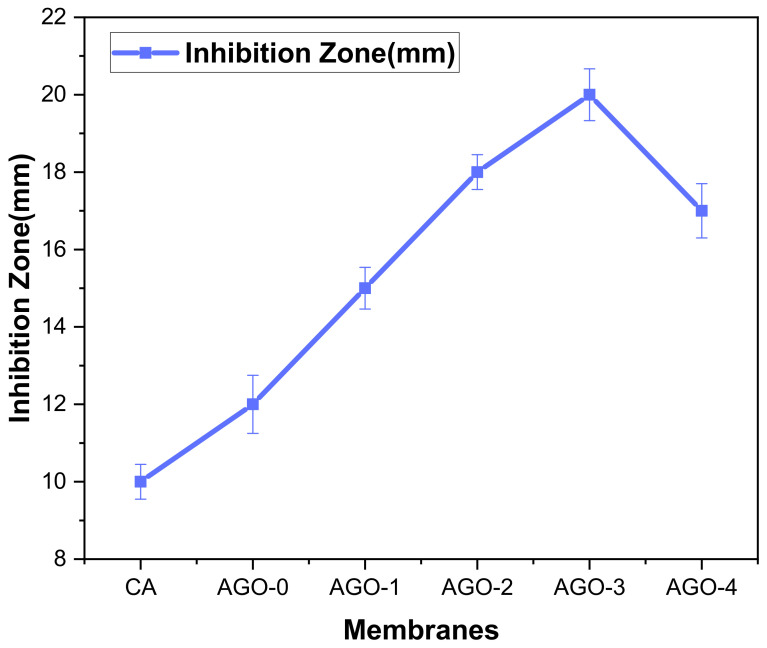
Antibacterial activity of AGO membranes.

**Figure 10:**
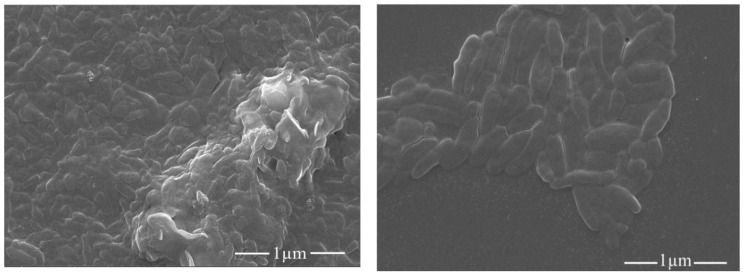
SEM images of pristine and AGO membrane.

The Antibacterial activity of AGO membranes was evaluated by calculating the inhibition zone, and results are shown in Figure 9. Results indicated that AGO membranes show significant growth inhibition toward bacteria, while with further addition, the antibacterial activity of the hybrid membrane decreased. It might be because the aggregation of the composite on the membrane surface and the viscosity of the casting solution during membrane fabrication increased [27]. AGO-3 membranes showed the highest antibacterial activity. Additionally, SEM images (Figure 10) of membranes also show that the number of bacteria on the surface of AGO membranes decreased as compared to that on the pristine membrane, which confirms the antibacterial activity of these fabricated membranes.

**Figure 11:**
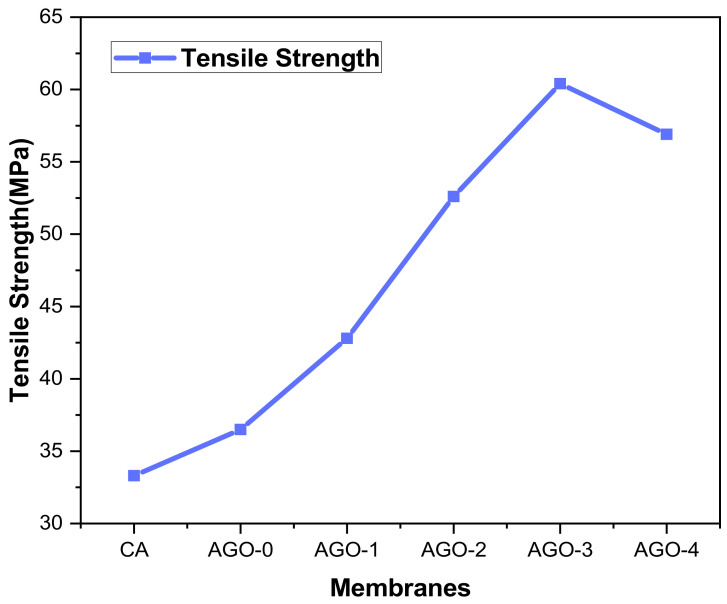
Tensile strength of AGO membranes.

The mechanical properties of AGO membranes were evaluated and are represented in Figure 11. Our results revealed that the tensile strength of AGO membranes increases with the incorporation of NH_2_–GO composites. This might be because of the strong interfacial bonding between the polymer chains and functional groups of composites. The amine group itself helps to interact with the polymer and causes great compatibility between the composite and polymer. While the further addition of composite decreases the tensile strength and so the thermal properties decreased. This could be due to the increase in porosity of the nanocomposite membrane resulting in a decrease in mechanical properties [30].

## 4. Conclusions

Aminated graphene oxide–based cellulose acetate membranes were fabricated via the phase inversion method to improve the hydrophilicity and antifouling properties of membranes. Structural characterization of the membrane indicates the incorporation of the composite successfully on the membrane surface. Hydrophilic groups of aminated graphene oxide improve the characteristics of cellulose acetate membranes. AGO membranes showed low contact angle, high water flux, and more thermal and mechanical stability. These fabricated membranes also have high antibacterial activity as compared to pristine cellulose acetate membranes, which makes them promising and efficient membranes.

## Figures and Tables

**Table 1:** Concentrations of different AGO membranes.

Membrane	CA (wt. %)	THF	GO	NH_2_–GO
CA	15%	85%	-	-
AGO-0	15%	84.9%	0.10%	-
AGO-1	15%	84.98%	-	0.02%
AGO-2	15%	84.95%	-	0.05%
AGO-3	15%	84.9%	-	0.10%
AGO-4	15%	84.85%	-	0.15%

## Data Availability

Not applicable.

## References

[1] Yin J., Deng B. (2015). Polymer-matrix nanocomposite membranes for water treatment. J. Membr. Sci..

[2] Pendergast M.M., Hoek E.M. (2011). A review of water treatment membrane nanotechnologies. Energy Environ. Sci..

[3] He Y., Xu L., Feng X., Zhao Y., Chen L. (2017). Dopamine-induced nonionic polymer coatings for significantly enhancing separation and antifouling properties of polymer membranes: Codeposition versus sequential deposition. J. Membr. Sci..

[4] Mohan T., Mohan T., Kargl R., Tradt K.E., Kulterer M.R., Braćić M., Hribernik S., Stana-Kleinschek K., Ribitsch V. (2015). Antifouling coating of cellulose acetate thin films with polysaccharide multilayers. Carbohydr. Polym..

[5] Vetrivel S., Saraswathi M.S.A., Rana D., Nagendran A. (2018). Fabrication of cellulose acetate nanocomposite membranes using 2D layered nanomaterials for macromolecular separation. Int. J. Biol. Macromol..

[6] Park H.B., Kamcev J., Robeson L.M., Elimelech M., Freeman B.D. (2017). Maximizing the right stuff: The trade-off between membrane permeability and selectivity. Science.

[7] Kwan S.E., Bar-Zeev E., Elimelech M. (2015). Biofouling in forward osmosis and reverse osmosis: Measurements and mechanisms. J. Membr. Sci..

[8] Yu C., Wu J., Zin G., Di Luccio M., Wen D., Li Q. (2018). d-Tyrosine loaded nanocomposite membranes for environmental-friendly, long-term biofouling control. Water Res..

[9] Faria A.F., Liu C., Xie M., Perreault F., Nghiem L.D., Ma J., Elimelech M. (2017). Thin-film composite forward osmosis membranes functionalized with graphene oxide–silver nanocomposites for biofouling control. J. Membr. Sci..

[10] Vetrivel S., Saraswathi M.S.A., Rana D., Divya K., Nagendran A. (2018). Cellulose acetate composite membranes tailored with exfoliated tungsten disulfide nanosheets: Permeation characteristics and antifouling ability. Int. J. Biol. Macromol..

[11] Banerjee I., Pangule R.C., Kane R.S. (2011). Antifouling coatings: Recent developments in the design of surfaces that prevent fouling by proteins, bacteria, and marine organisms. J. Adv. Mater..

[12] Sun W., Shi J., Chen C., Li N., Xu Z., Li J., Lv H., Qian X., Zhao L. (2018). A review on organic–inorganic hybrid nanocomposite membranes: A versatile tool to overcome the barriers of forward osmosis. RSC Adv..

[13] Zinadini S., Rostami S., Vatanpour V., Jalilian E., Rostami S., Vatanpour V., Jalilian E. (2017). Preparation of antibiofouling polyethersulfone mixed matrix NF membrane using photocatalytic activity of ZnO/MWCNTs nanocomposite. J. Membr. Sci..

[14] Madaeni S., Enayati E., Vatanpour V. (2011). Separation of nitrogen and oxygen gases by polymeric membrane embedded with magnetic nano-particle. Polym. Adv. Technol..

[15] Liang S., Xiao K., Mo Y., Huang X. (2012). A novel ZnO nanoparticle blended polyvinylidene fluoride membrane for anti-irreversible fouling. J. Membr. Sci..

[16] Derbali Z., Fahs A., Chailan J.F., Ferrari I.V., Di Vona M.L., Knauth P. (2017). Composite anion exchange membranes with functionalized hydrophilic or hydrophobic titanium dioxide. Int. J. Hydrog. Energy..

[17] Pandey R.P., Shukla G., Manohar M., Shahi V.K. (2017). Graphene oxide based nanohybrid proton exchange membranes for fuel cell applications: An overview. Adv. Colloid Interface Sci..

[18] Zhao C., Xu X., Chen J., Wang G., Yang F. (2014). Highly effective antifouling performance of PVDF/graphene oxide composite membrane in membrane bioreactor (MBR) system. Desalination.

[19] Yin J., Zhu G., Deng B. (2016). Graphene oxide (GO) enhanced polyamide (PA) thin-film nanocomposite (TFN) membrane for water purification. Desalination.

[20] Ederer J., Janoš P., Ecorchard P., Tolasz J., Štengl V., Beneš H., Perchacz M., Pop-Georgievski O. (2017). Determination of amino groups on functionalized graphene oxide for polyurethane nanomaterials: XPS quantitation vs. functional speciation. RSC Adv..

[21] Li X., Zhao C., Yang M., Yang B., Hou D., Wang T. (2017). Reduced graphene oxide-NH2 modified low pressure nanofiltration composite hollow fiber membranes with improved water flux and antifouling capabilities. Appl. Surf. Sci..

[22] Kumar M., Sreedhar N., Jaoude M.A., Arafat H.A., Sreedhar N., Jaoude M.A., Arafat H.A. (2019). High-flux, antifouling hydrophilized ultrafiltration membranes with tunable charge density combining sulfonated poly (ether sulfone) and aminated graphene oxide nanohybrid. ACS Appl. Mater. Interfaces.

[23] Khan S.B., Alamry K.A., Bifari E.N., Asiri A.M., Yasir M., Gzara L., Ahmad R.Z. (2015). Assessment of antibacterial cellulose nanocomposites for water permeability and salt rejection. J. Ind. Eng. Chem..

[24] Lai L., Chen L., Zhan D., Sun L., Liu J., Lim S.H., Poh C.K., Shen Z., Lin J. (2011). One-step synthesis of NH2-graphene from in situ graphene-oxide reduction and its improved electrochemical properties. Carbon.

[25] Li F., Li Y., Chung T.S., Kawi S. (2010). Facilitated transport by hybrid POSS^®^–Matrimid^®^–Zn^2+^ nanocomposite membranes for the separation of natural gas. J. Membr. Sci..

[26] Ge B.S., Wang T., Sun H.X., Gao W., Zhao H.R. (2018). Preparation of mixed matrix membranes based on polyimide and aminated graphene oxide for CO2 separation. Polym. Adv. Technol..

[27] Zhang H., Li B., Pan J., Qi Y., Shen J., Gao C., Van der Bruggen B. (2017). Carboxyl-functionalized graphene oxide polyamide nanofiltration membrane for desalination of dye solutions containing monovalent salt. J. Membr. Sci..

[28] Karimipour H., Shahbazi A., Vatanpour V. (2021). Fouling decline and retention increase of polyethersulfone membrane by incorporating melamine-based dendrimer amine functionalized graphene oxide nanosheets (GO/MDA). J. Environ. Chem. Eng..

[29] Xu H., Ding M., Liu S., Li Y., Shen Z., Wang K. (2017). Preparation and characterization of novel polysulphone hybrid ultrafiltration membranes blended with N-doped GO/TiO2 nanocomposites. Polym. J..

[30] Gzara L., Rehan Z.A., Khan S.B., Alamry K.A., Albeirutty M.H., El-Shahawi M.S., Rashid M.I., Figoli A., Drioli E., Asiri A.M. (2016). Preparation and characterization of PES-cobalt nanocomposite membranes with enhanced anti-fouling properties and performances. J. Taiwan Inst. Chem. Eng..

